# Multifunctional Gold Nanoparticles Overcome MicroRNA Regulatory Network Mediated-Multidrug Resistant Leukemia

**DOI:** 10.1038/s41598-019-41866-y

**Published:** 2019-03-29

**Authors:** Rong Deng, Bai Ji, Hongliang Yu, Wei Bao, Zhuoqi Yang, Ying Yu, Yahan Cui, Yangyang Du, Meiyu Song, Shujun Liu, Kamel Meguellati, Fei Yan

**Affiliations:** 1 State Key Laboratory of Inorganic Synthesis and Preparative Chemistry, International Research Center for Chemistry-Medicine Joint Innovation, College of Chemistry, Jilin University, 2699 Qianjin Street, Changchun, 130012 China; 2 International Joint Research Laboratory of Nano-Micro Architecture Chemistry (NMAC), College of Chemistry, Jilin University, 2699 Qianjin Street, Changchun, 130012 China; 3 Department of Hepatobiliary and Pancreatic Surgery, the First Hospital of Jilin University, Changchun, 130021 China; 4 The Hormel Institute, University of Minnesota, 801 16th Avenue NE, Austin, MN 55912 USA

## Abstract

Resistance to chemotherapy and molecularly targeted therapies is a major problem in current leukemia treatments. Here, we investigated cross-talk between the miR-221 network and P-glycoprotein (P-gp) in doxorubicin-induced drug resistance of leukemia cells. Multifunctional gold nanoparticles were designed and synthesized to co-deliver three anticancer agents, AS1411, doxorubicin and anti-221, for improving leukemia treatment efficacy. These nanoparticles significantly inhibited the proliferation and clonogenic potential, and induced apoptosis of drug-resistant leukemia cells. The decreased growth of drug-resistant cells induced by these nanoparticles was associated with marked downregulation of miR-221 and DNMT1, leading to restored p27kip1 and p15ink4b tumor suppressor expression, as well as miR-221-mediated reduction of P-gp expression. Finally, primary blasts derived from leukemia patients experiencing chemoresistant relapse that were exposed to these nanoparticles were sensitized to doxorubicin, as evidenced by suppression of leukemic cell growth and a significant reduction of the doxorubicin IC50 value. Our findings provide proof of concept that this novel drug delivery system can precisely reverse the multidrug resistant leukemia phenotype based on preclinical models of leukemia, providing the framework for future clinical trials aimed at overcoming drug resistance and improving patient outcome.

## Introduction

Leukemia is a heterogeneous form of cancer characterized by the abnormal growth of leukocytes due to genetic aberrations and mutations. Chemotherapeutic agents such as doxorubicin are frequently used as the first-line therapy for patients with leukemia, but the effectiveness of these agents is limited by drug resistance^1^. Although the precise mechanisms underlying the drug-resistant phenotype remain elusive, accumulating evidence suggests that the acquisition of drug resistance involves a complex and multifactorial process, including increased drug efflux^2^, mutations of the drug target^3^, activation of alternative signaling pathways^4^, and epigenetic mechanisms^5,6^. The development of multidrug resistance (MDR) is a major obstacle to improving treatment outcomes and extending overall survival in leukemia^7^. A key mechanism associated with MDR is the chemotherapeutic agent- induced overexpression of P-glycoprotein (P-gp), a plasma membrane drug efflux pump that possesses high anticancer agent transport efficiency, leading to a reduction in complete remission rates during cancer therapy. Therefore, inhibition of P-gp expression or its drug efflux function has been an important strategy to overcome MDR^8,9^.

Drug delivery strategies based on nanotechnology can significantly improve target specificity and drug stability for overcoming MDR, especially in solid tumors, because the nanoparticles easily and passively accumulate due to the enhanced permeability and retention (EPR) effect^10^. Ye *et al*. reported a new therapeutic strategy using a cascade amplification drug release system to overcome MDR in solid tumors^11^. The Chen group described a glutathione-responsive polymer prodrug of SO_2_ acting as a stimuli-responsive nanocarrier to co-deliver DOX, which can efficiently inhibit the proliferation of MCF-7ADR cells^12^. Zhang *et al*. assembled a three-way junction nanoparticle to target HER2-overexpressing human breast cancer via a new drug resistant mechanism for overcoming tamoxifen resistance^13^. Nevertheless, it is still a challenge to develop nanotherapies for leukemia because it is difficult for nanocarriers to accumulate passively by the EPR effect and target leukemia cells^14,15^. Thus, development of novel nanoplatforms based on active-targeting strategies which rely on a better understanding of the molecular pathogenesis and drug response mechanisms, is especially important in leukemia. Liu *et al*. reported that a transferrin-conjugated nanoparticle system efficiently delivered synthesized miR-29b mimics to AML blasts and improved the antileukemic activity of decitabine by priming AML cells through downregulation of DNMTs, CDK6, SP1, KIT, and FLT3 genes, which are frequently overexpressed or mutated in AML^16^. We previously described a high-density lipoprotein-coated AuNP therapeutic system for acute myeloid leukemia to deliver a small molecule that selectively inhibits AML-promoting factor fatty acid-binding protein 4^17,18^.

MicroRNAs (miRNAs) are small non-coding RNA molecules that act as regulators of their target genes by either degrading mRNA or inhibiting their translation. Targeting microRNA networks is a promising strategy to overcome drug resistance in human cancer^19,20^. Among the miRNAs involved in the development of drug resistance, miR-221 plays a key role, since inhibition of miR-221 has been shown to counteract resistance to anticancer agents in a variety of cancers^21,22^. Although miRNA or their inhibitors showed promising results in phase I or II clinical trials, the efficient delivery of miRNAs to their target tissues is a major problem for translating miRNA therapies to the clinic. Recently, we showed that inhibition of oncogenic miR-221 with a nuclear-targeted gold nanoparticle induced significant anti-leukemogenetic activity in AML by targeting epigenetic pathways involved in the regulation of cell proliferation and survival, and these nanoparticles proved to be safe and effective for systemic delivery in mice^23^.

**Figure 1:**
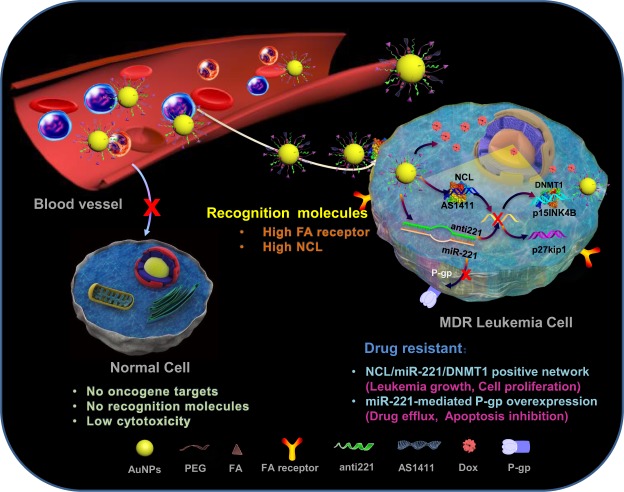
Illustration of multifunctional gold nanoparticles to overcome P-gp-mediated multidrug-resistance in leukemia cells by targeting the miR-221 network.

Herein, we describe a multi-target drug delivery system that can specifically target multidrug-resistant leukemia cells based on mechanistic crosslink between the miR-221 network and P-gp. As shown in Fig. 1, 40 nm gold nanoparticles were functionalized via thiol modifications at the 5′ end of the nucleotides of anti-221, the miR-221 inhibitor, and the AS1411 aptamer, a guanine-rich oligonucleotide that can form a stable G-quadruplex structure to specifically target nucleolin, which is overexpressed on the leukemia cell surface. Multi-functional nanoparticles were loaded with DOX via noncovalent intercalation between DOX and consecutive CG base pairs designed within the extended region at the 3′ end of AS1411. In addition, since high folate (FA) receptor expression has been reported in leukemia cells^24,25^, we functionalized the nanoparticles with FA-conjugated PEG to achieve an efficient, targeted and biocompatible delivery. Gold nanoparticles can easily enter mammalian cells via the endocytosis pathway^26^. In addition, some publications have demonstrated that the AS1411 aptamer can also improve the cellular uptake of nanoparticles^27,28^. AS1411 and anti-221 are released in leukemia cells because the gold-thiolate bonds (Au-S) between AS1411/anti-221 and AuNPs are broken by place-exchange reactions under the reductive cytosolic conditions found in cancer cells^29,30^. Thus, by specific recognition of AS1411 and FA, the nanocarriers enter the target resistant leukemia cells, whereas nonspecific uptake by normal cells is minimized due to the absence of cell membrane receptors and intracellular targets. Subsequently, anti-221 and AS1411 synergistically suppress endogenous miR-221 and NCL function in the NCL/miR-221 pathway, which contributes to leukemogenesis. DOX is released from AuNP-AS1411 and interacts with DNA for inhibition of macromolecular biosynthesis, synergistically blocking cell proliferation and inducing cellular apoptosis together with anti-221 and AS1411. More importantly, P-gp downregulation by anti-221 makes the drug-resistant cells more sensitive, leading to enhanced chemotherapeutic efficacy. Therefore, maximum therapeutic efficacy with minimum side effects are achieved with multifunctional nanoparticles, offering preclinical proof of concept for this novel targeted drug delivery carrier system for cancer therapy.

**Figure 2:**
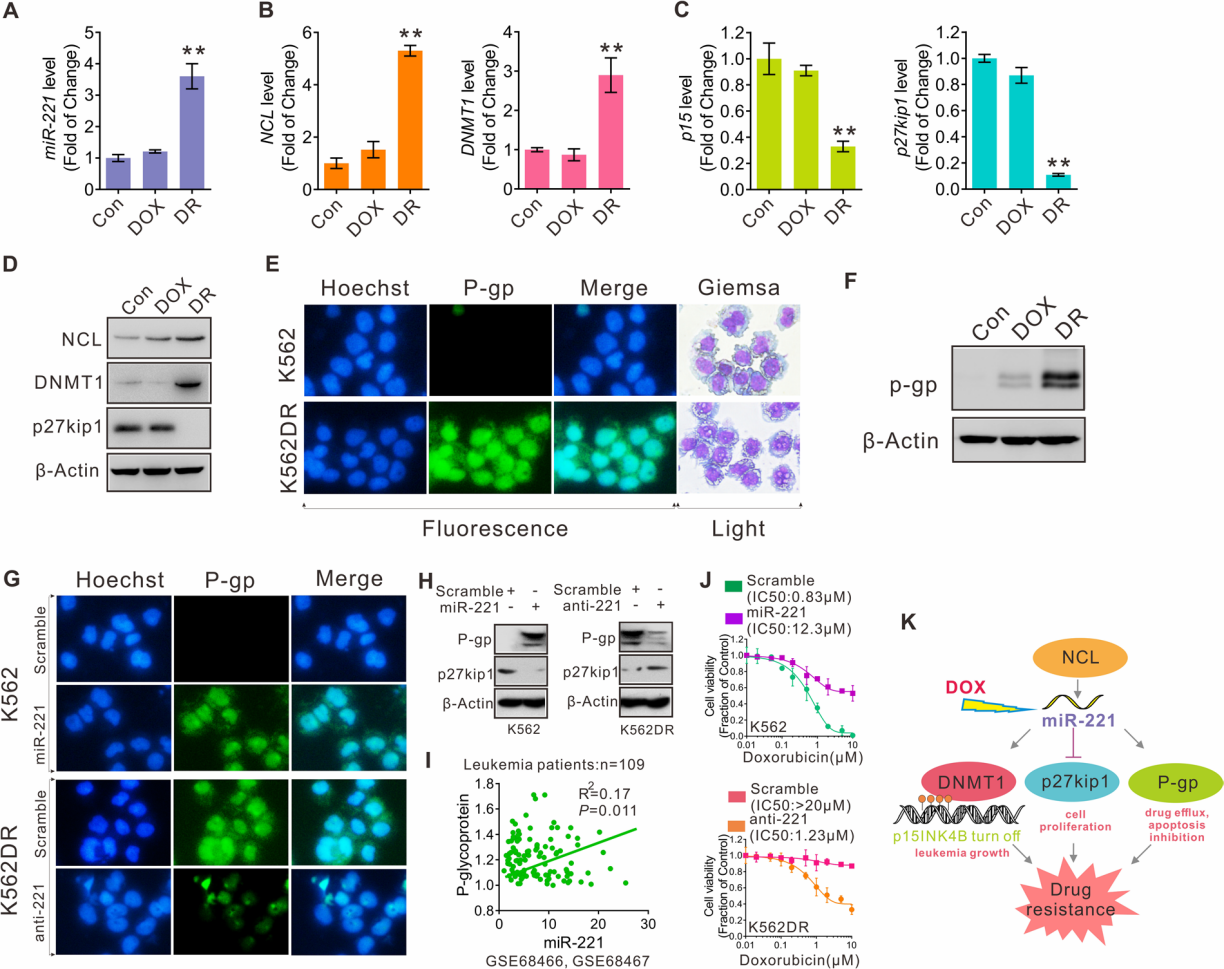
Regulatory and functional interactions between the miR-221 network and P-gp in multidrug-resistant leukemia cells. (**A**–**C**) qPCR measuring the expression levels of (**A**) miR-221, (**B**) NCL, DNMT1 and (**C**) p15ink4b, p27kip1 in K562, DOX-treated K562 (10 µM, 24 h) and K562DR cells. Data represent three independent experiments and are expressed as the mean ± SD; **P < 0.01 (**D**) Western blot showing NCL, DNMT1 and p27kip1 protein expression in K562, DOX-treated K562 (10 µM, 24 h) and K562DR cells. (**E**) Immunofluorescence assay showing P-gp expression in K562 and K562DR cells (blue, hoechst; green, P-gp; light, giemsa staining of leukemia cells). (**F**) Western blot showing P-gp protein expression in K562, DOX-treated K562 (10 µM, 24 h) and K562DR cells. (**G**) Immunofluorescence assay showing P-gp expression in K562 cells transfected with miR-221 or a scrambled control and in K562DR cells transfected with anti-221 or a scrambled control (blue, hoechst; green, P-gp). (**H**) Western blot showing p27kip1 and P-gp protein expression in K562 and K562DR cells transfected with miR-221, anti-221 or a scrambled control. (**I**) Spearman correlation analysis of miR-221 and P-gp RNA levels in AML patients (109 AML patients, GEO database GSE68466 and GSE68467). (**J**) K562 (above) and K562DR (below) cells were transfected with miR-221, anti-221 or scrambled control for 6 hours, treated with doxorubicin (0 to 10 µM) for an additional 72 hours and cellular viability was measured with the CCK-8 assay. (**K**) Summary diagram describes regulatory and functional interactions between the miR-221 network and P-gp in multidrug-resistant leukemia cells.

## Results and Discussion

### Mechanistic cross-talk between the miR-221 network and P-gp promotes leukemic cell resistance to doxorubicin

Resistance of leukemia cells to anticancer agents is a major cause of chemotherapeutic failure, indicating the need for new treatment options based on new mechanistic findings. To dissect the underlying molecular rules, we developed a model of leukemia resistance to DOX by treating K562 cells with increasing doses of DOX for > 2 months to select DOX-resistant cells (K562DR). The final concentration was 10 µM, which exerts sufficiently inhibitory activity and is within the range of clinically attainable levels. To characterize drug resistant phenotypes, we measured cell variability, colony forming ability and apoptosis using the CCK-8 assay (Fig. S1A), colony assay (Fig. S1B,C) and Annexin V-FITC/PI (Fig. S1D). Compared with parental cells (K562), K562DR cells were significantly more resistant to DOX treatment. MiR-221 plays an important role in the modulation of the response to anti-cancer agents through the regulation of key oncogenic factors such as TP53, PTEN and p27kip1^31,32^. Our previous findings demonstrated that the miR-221-mediated epigenetic network contributes to AML cell growth^23^. MiR-221 also plays a key role in the development of drug resistance through the inhibition of caspase-3 mediated apoptosis^22^. Based on these findings, we hypothesized that miR-221 could play a mechanistic role in multidrug-resistant leukemia cells. As shown in Fig. 2A, miR-221 levels in K562DR cells were significantly higher than in K562 cells. Our previous studies showed that miR-221 overexpression could potentially act in parallel with the NCL/DNMT1 axis to promote leukemic cell growth. To investigate whether the NCL/miR-221/DNMT1 pathway was involved in the multidrug-resistant phenotype of leukemic cells, we performed qPCR and Western blot assays to measure NCL and DNMT1 RNA or protein levels in K562DR cells. Indeed, we found that NCL and DNMT1 were overexpressed in K562DR cells (Fig. 2B), resulting in inactivity of the downstream tumor suppressors p27kip1 and p15ink4b (Fig. 2C). Western blot assays confirmed these results at the protein level (Fig. 2D). Overexpression of P-glycoprotein (P-gp), a drug efflux pump at the plasma membrane, is the most frequently observed molecular cause of multidrug resistance^33^. As shown in Fig. 2E,F, we observed high expression levels of p-GP in K562DR compared with parental cells. We didn’t see any significant differences in DOX-treated K562 cells (10 µM, 24 h) in terms of miR-221, NCL, DNMT1, p15ink4b or p27kip1 expression levels, suggesting no direct effect of DOX treatment on the expression of the analyzed target molecules. P-gp can be regulated by a variety of miRNAs^34,35^, but considering the overexpression of miR-221 in K562DR cells, we predicted there could be some specific interactions between miR-221 and P-gp. We hypothesized that miR-221 could regulate P-gp expression during the development of multidrug resistance in leukemia. To investigate the regulatory interactions between miR-221 and P-gp, we employed a “gain and loss of function” strategy to specifically manipulate miR-221 expression in K562DR or K562 parental cells. Western blot and immunofluorescence assays revealed that anti-miR-triggered miR-221 depletion resulted in downregulation of P-gp, whereas miR-221 overexpression resulted in the upregulation of P-gp (Fig. 2G,H). To examine whether overexpressed miR-221 in leukemia patients was associated with P-gp, GEO data (GSE68466 and GSE68467) of miR-221 and P-gp mRNA expression were analyzed by Spearman correlation analysis. We found that miR-221 and P-gp showed a significant positive correlation (Fig. 2I) in patients with leukemia. Moreover, miR-221 overexpression in K562 parental cells made the cells more resistant to DOX, resulting in notably higher IC50 values (scrambled, IC50 = 0.83 µM; miR-221, IC50 = 12.3 µM). In contrast, miR-221 downmodulation rendered resistant cells more sensitive to DOX, as evidenced by the decreased cell proliferation rates and lower IC50 values (scrambled, IC50 > 20 µM; anti-221, IC50 = 1.23 µM, Fig. 2J). Taken together, these findings support the notion that regulatory and functional interactions occur between the miR-221 network and P-gp in multidrug-resistant leukemia cells: DOX-induced miR-221 overexpression, occurring upon activation of DNMT1 as well as P-gp, leads to p27kip1 and p15ink4b downregulation, which contributes to leukemic cell growth, apoptosis inhibition and drug resistance (Fig. 2K).

### Preparation of gold nanoparticles to deliver AS1411, DOX, and anti-221

**Figure 3:**
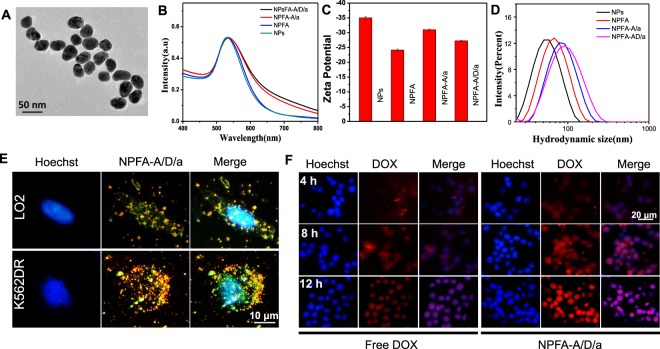
Characterization of the nanoparticles. (**A**) TEM images (scale bar = 50 nm) (**B**) Absorption spectra, (**C**) Zeta potential, and (**D**) Size distribution of the gold nanoparticles. (**E**) Fluorescence and dark-field imaging showing NPsFA-AS1411/DOX/a221 recognition by K562DR and LO2 cells (**F**) Fluorescence imaging showing K562DR cells treated with free DOX or NPsFA-AS1411/DOX/a221 for 4, 8 and 12 hours (blue, hoechst; red, DOX).

Given that the miR-221 network was associated with P-gp activation in DOX-resistant leukemia cells, it seemed logical to design a multiple-drug delivery system. We designed a multiple target co-delivery system consisting of anti-221, AS1411 and DOX (NPsFA-AS1411/DOX/a221). It has been reported that aptamers with GC sequences can be used as DOX carriers by noncovalent intercalation^36,37^. Here, consecutive CG base pairs were designed within the extended region at the 3′ end of AS1411 to ensure high drug-loading capacity. AS1411 is a 26-mer G-rich DNA oligonucleotide that forms G-quadruplex-containing structures and binds specifically to the external domain of NCL. AS1411 is the first DNA aptamer to reach phase I and II clinical trials for the potential treatment of cancer, including leukemia^38,39^. The interactions between AS1411 and DOX were demonstrated by measuring the fluorescence spectra of DOX. With increasing AS1411 concentrations, the intensity of DOX gradually decreased, as more DOX became incorporated in the GC framework (Fig. S2). To enhance the AS1411, anti-221 and DOX loading capacity, we used gold nanoparticles with a size of about 40 nm (Fig. 3A). The process of synthesis of this multifunctional gold nanoparticle was characterized by changes in the ultraviolet spectra, zeta potential and size distribution. As shown in Fig. 3B–D, significant changes can be seen directly, demonstrating successful functionalization of the gold nanoparticles. The AS1411, anti-221 and DOX amounts on the surface of the gold nanoparticles were calculated by the standard curve method, and details are shown in Fig. S3. The stability of NPsFA-AS1411/DOX/a221 was demonstrated by analyzing changes in the absorption spectra after incubating in PBS and serum buffer for 3 h, and observing no apparent peak movement (Fig. S4). These results suggest that the nanoparticles are stable before entering cells. Furthermore, the FA modification on the surface of the nanoparticles allows their enhanced recognition by tumor cells, and the fluorescence signal was mostly seen in K562DR cells treated with NPsFA-AS1411/DOX/a221 (Fig. 3E). K562DR cells treated with NPsFA-AS1411/DOX/a221 displayed a stronger signal than cells treated with free DOX, indicating successful drug delivery by NPsFA-AS1411/DOX/a221, high intracellular DOX release and accumulation, and effective reduction of cytotoxicity associated with combined AS1411 and anti-221 delivery for overcoming DOX resistance (Fig. 3F).

### NPsFA-AS1411/DOX/a221 blocks proliferation, colony formation, and induces apoptosis of K562DR cells

**Figure 4:**
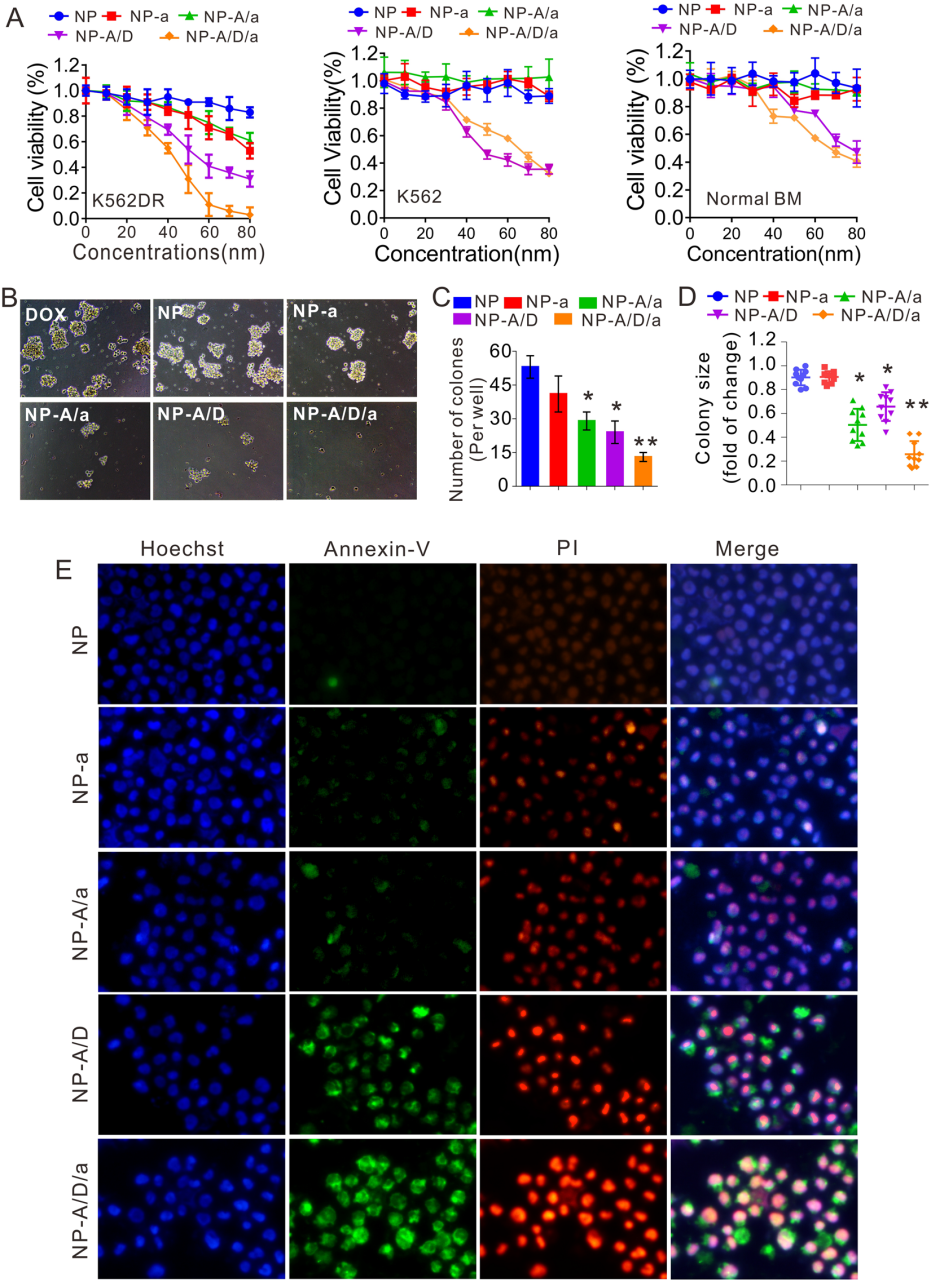
Inhibition of proliferation, colony formation and induction of apoptosis of drug-resistant K562 leukemic cells. (**A**) CCK-8 assays measuring proliferation of K562DR, K562 and normal bone marrow cells treated with different doses of the indicated nanoparticles for 48 hours. Data represent three independent experiments, each conducted in triplicate. (**B**–**D**) K562DR cells were treated with various nanoparticles and subjected to colony-forming assays. Representative clones are shown (**B**). Graphs show colony numbers from three independent experiments (**C**) and colony sizes, calculated by measuring ten clones (**D**). (**E**) Fluorescence assay measuring apoptosis of K562DR cells treated with the indicated nanoparticles for 24 hours (blue, hoechst; green, Annexin-V; red, PI). Data represent the mean ± SD; *P < 0.05, **P < 0.01. Note: NP, NP-a, NP-A/a, NP-A/D, and NP-A/D/a abbreviations indicate NPsFA, NPsFA-a221, NPsFA-AS1411/a221, NPsFA-AS1411/DOX and NPsFA-AS1411/DOX/a221, respectively.

Based on the observed targeting specificity of NPsFA-AS1411/DOX/a221 towards K562DR cells, its properties were further evaluated. First, we treated K562DR cells with increasing concentrations of nanoparticles for 48 hours and measured cell proliferation with the CCK-8 assay. As demonstrated in Fig. 4A (left), NPsFA-AS1411/DOX/a221 induced a dose-dependent inhibition of cell growth (0–80 nM, final concentrations of oligonucleotides) when compared with NPsFA, NPsFA-a221, NPsFA-AS1411/a221 and NPsFA-AS1411/DOX. Interestingly, K562 parental cells were insensitive to the nanoparticles (Fig. 4A, middle), which is consistent with their lower target gene expression and suggests that the improved therapeutic effect of NPsFA-AS1411/DOX/a221 depends on the miR-221 network in drug-resistant cells. Notably, no obvious cytotoxicity was seen when LO2 cells (normal human cells, Fig. S5) and bone marrow cells isolated from normal mice (Fig. 4A, right) were treated with NPsFA, NPsFA-a221 or NPsFA-AS1411. DOX-loaded nanoparticles (NPsFA-AS1411/DOX and NPsFA-AS1411/DOX/a221) inhibited cell growth (K562 and normal bone marrow cells) at high concentrations due to the nonspecific cytotoxic effect of doxorubicin. To further examine the inhibitory effect of the nanoparticles, we performed colony formation and Annexin V-FITC/PI apoptosis assays using 50 nM (final concentration of oligonucleotides) of NPsFA, NPsFA-a221, NPsFA-AS1411/a221, NPsFA-AS1411/DOX and NPsFA-AS1411/DOX/a221. Paralleling the cell proliferation results, NPsFA-AS1411/DOX/a221 was the most potent inhibitor of K562DR cells, as indicated by reduced colony number and size (Fig. 4B–D) and the highest induction of apoptosis (Fig. 4E).

### NPsFA-AS1411/DOX/a221 counteracts miR-221/p-GP-dependent multidrug resistance in leukemia

**Figure 5:**
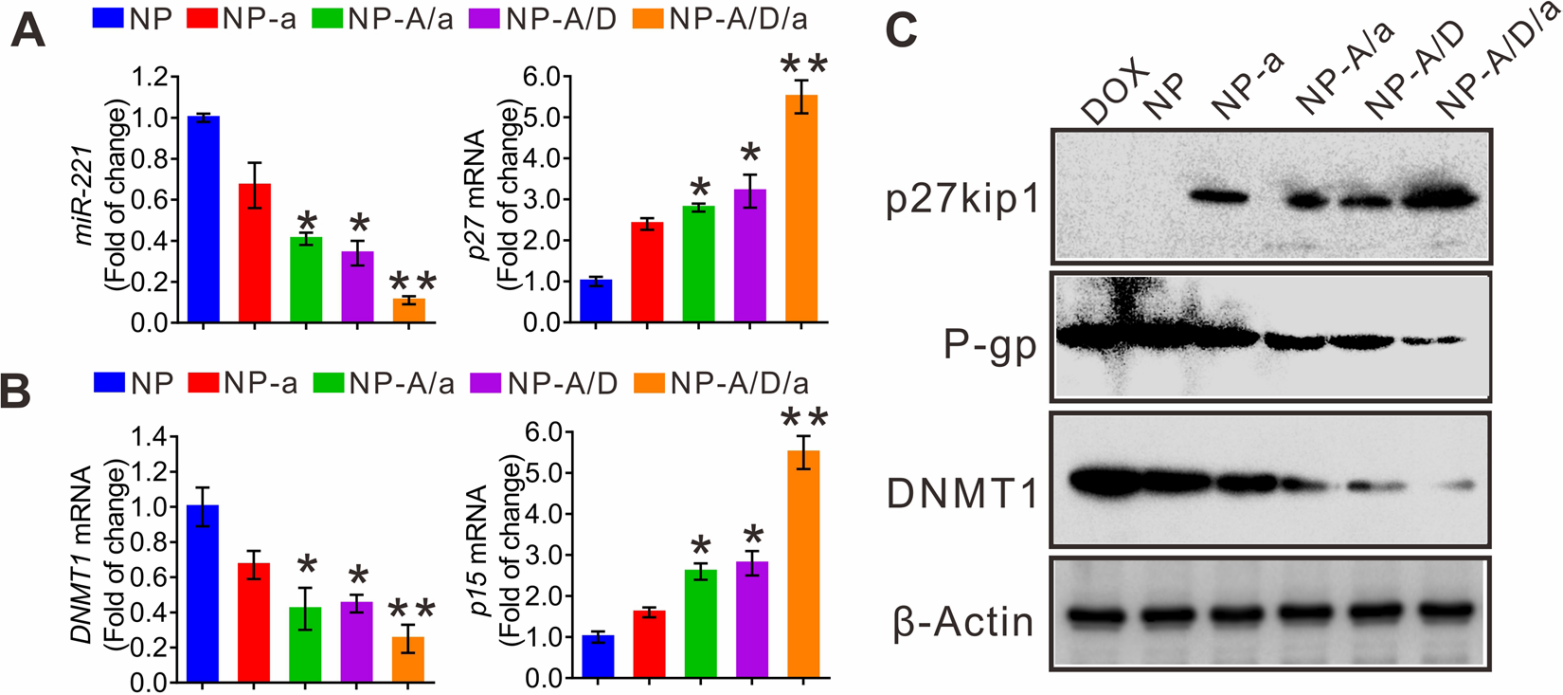
NPsFA-AS1411/DOX/a221 cooperatively downregulates the miR-221 pathway and modulates P-gp levels in K562DR cells. (**A**,**B**) qPCR measuring RNA levels of miR-221, p27kip1 (**A**) and DNMT1, p15ink4b (**B**) in K562DR cells treated with the indicated nanoparticles for 48 hours. Data represent three independent experiments and are expressed as the mean ± SD; *P < 0.05, **P < 0.01. (**C**) Western blot analyzing p27kip1, P-gp and DNMT1 protein expression levels in K562DR cells treated with the indicated nanoparticles for 48 hours. Note: NP, NP-a, NP-A/a, NP-A/D, and NP-A/D/a abbreviations indicate NPsFA, NPsFA-a221, NPsFA-AS1411/a221, NPsFA-AS1411/DOX and NPsFA-AS1411/DOX/a221, respectively.

To explore whether the inhibition of K562DR cell growth by nanoparticles was driven by the miR-221/P-gp network, we measured miR-221, DNMT1, p27kip1 and p15ink4b RNA or protein levels in the various treatment groups. As shown in Fig. 5, we found that NPsFA-AS1411/DOX/a221 treatment synergistically suppressed miR-221 (Fig. 5A) and DNMT1 (both RNA and protein levels, Fig. 5B,C) expression, resulting in higher activity of their targets p27kip1 (both RNA and protein levels, Fig. 5A,C) and p15ink4b (RNA level, Fig. 5B). In particular, P-gp protein expression showed the strongest downregulation by NPsFA-AS1411/DOX/a221 when compared with other nanoparticles or free DOX (Fig. 5C). Collectively, these data suggest that the multifunctional gold nanoparticles synergistically block leukemia resistance to DOX through the miR-221 pathway, associated with P-gp suppression.

### NPsFA-AS1411/DOX/a221 sensitizes primary blasts from leukemia patients experiencing chemoresistant relapse to doxorubicin

**Figure 6:**
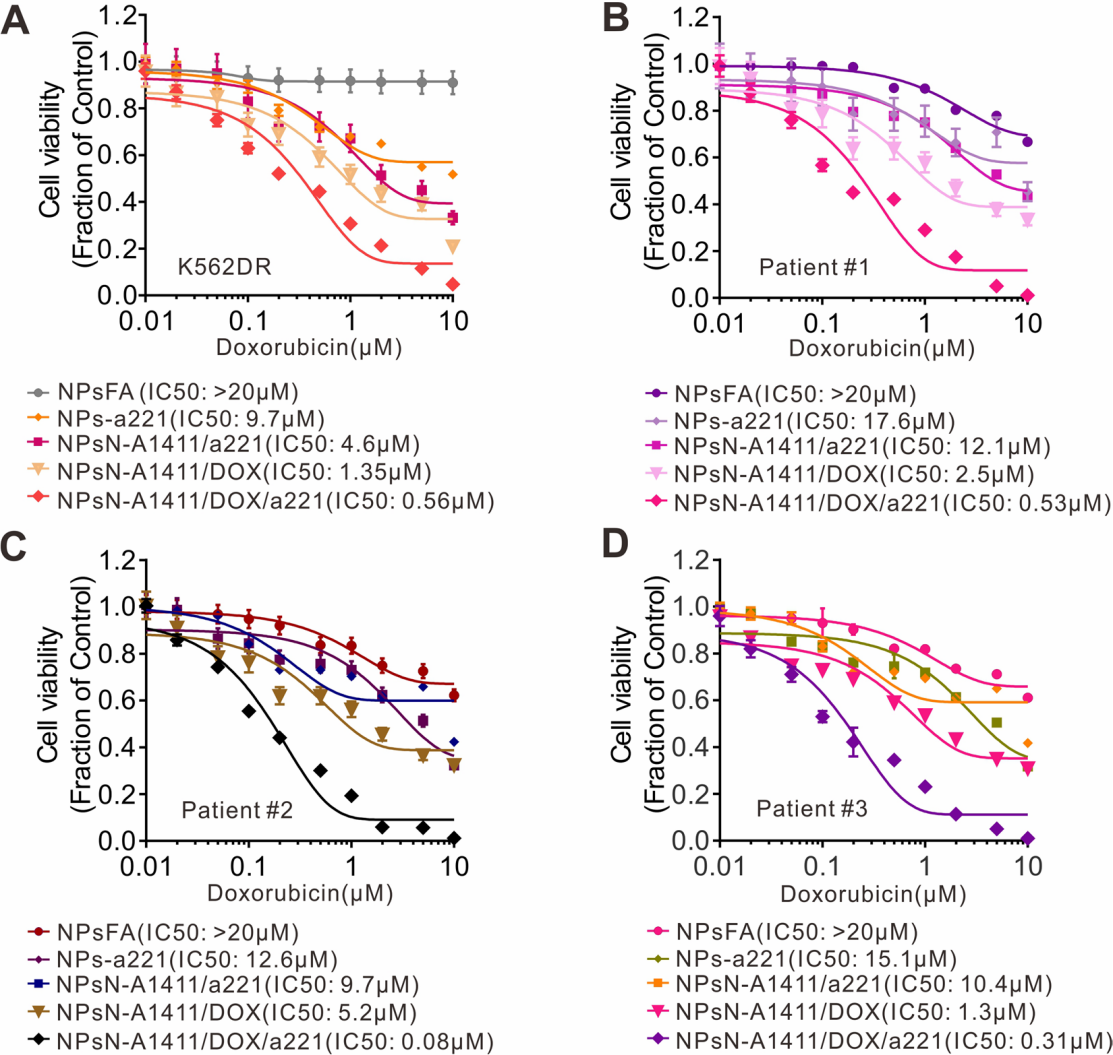
NPsFA-A1411/DOX/a221 nanoparticles sensitize leukemia cells that are resistant to chemotherapy. (**A**) CCK-8 assays measuring proliferation of K562DR or (**B**–**D**) three primary cells derived from AML patients experiencing chemoresistant relapse and treated with the indicated nanoparticles for 24 hours, followed by incubation with different doses of doxorubicin for an extra 48 hours. Data represent 3 independent experiments, each conducted in triplicate.

Finally, to test the effect of the nanoparticles on DOX resistant cells, we treated K562DR cells that had been incubated with DOX with the nanoparticles, as shown in Fig. 6A. The nanoparticles significantly sensitized K562DR cells to DOX (NPsFA, IC50 > 20 µM; NPsFA-a221, IC50 = 9.7 µM; NPsFA-A1411/a221, IC50 = 4.6 µM; NPsFA-A1411/DOX, IC50 = 1.35 µM; NPsFA-A1411/DOX/a221, IC50 = 0.56 µM). More importantly, to explore the possible clinical application of the nanoparticles as a MDR counteracting agent, primary blasts from leukemia patients experiencing chemoresistant relapse (n = 3) were treated with the nanoparticles for 72 hours. The patient information is shown in Table 1. Consistent with their ability to strongly disrupt K562DR cell growth, the NPsFA-AS1411/DOX/a221 nanoparticles markedly suppressed leukemia cell proliferation, showing the lowest IC50 values when compared with other nanoparticles (Fig. 6B–D). These promising results indicate that NPsFA-A1411/DOX/a221 nanoparticles can significantly sensitize drug-resistant leukemia cells to DOX, as determined using both cell lines and primary cells, suggesting they could potentially be translated from the laboratory to the clinic.

**Table 1:** Clinical Characteristics of AML Patients.

Characteristic	Patient 1	Patient 2	Patient 3
Age	55	44	22
Sex	Male	Female	Female
WBC (×10^9^/L)	26.1	13.9	51
BM blasts (%)^a^	44.9	29.3	55.6
c-KIT mutation	No	No	No
FLT3 mutation	No	No	No
NPM1 mutation	No	No	No
FAB subtypes	M2	M2	M2
Relapse	Yes	Yes	Yes
Chemotherapy	Yes	Yes	Yes

In conclusion, we developed a novel multifunctional nanoparticle system to efficiently deliver AS1411, anti-221 and DOX to multidrug-resistant leukemia cells. Treatment with these nanoparticles increased DOX accumulation in resistant cells, downregulated the miR-221 network and its targets as well as P-gp, and showed antileukemic activity in both cell lines and primary leukemic cells. Our nanoparticle delivery approach is a promising new antileukemic strategy with clinical translation potential for overcoming multidrug-resistance.

## Methods

### Synthesis of gold nanoparticles

The citrate-stabilized gold nanoparticles (NPs) were synthesized according to a modified Turkevich method^40^. Briefly, 100 mL of 0.1% HAuCl_4_ solution was heated until it boiled and was reduced by adding 1 mL of 1.0% trisodium citrate. The mixture was then left heating until its color turned wine red, followed by cooling under a stream of water. The NPs were centrifuged at 8000 g for 10 min and resuspended in deionized water. They were characterized by means of UV-Vis spectroscopy, TEM and DLS (MALVERN Zetasizer Nano ZS).

### Preparation of FA-PEG-AuNPs

1 mM NH_2_-PEG-SH (MW = 2000) was added to a 10 nM solution of NPs with 1% of SDS and incubated for 24 h at room temperature. After removing excess NH_2_-PEG-SH by centrifugation at 6000 rpm for 10 min, the purified NH_2_-PEG-NPs were resuspended in deionized water. To prepare the FA-PEG-NPs, 1 mM of EDC and NHS were mixed and incubated for 30 min, then 1 mM of folic acid was added and the mixture shaken for an additional 30 min. After incubation with NH_2_-PEG-NPs for 12 h, excess folic acid, EDC and NHS were removed by two centrifugations (5000 rpm, 15 min) to obtain the purified FA-PEG-NPs.

### Preparation of DOX loaded NPsFA-AS1411/DOX/a221

15 mM of tris(2-carboxyethyl)phosphine (TCEP) was mixed with 50 μM of thiol-modified AS1411 and anti221. After 30 min, the mixture was added to the FA-PEG-NPs solution at a ratio of 200:1 (mol/mol). 2 M NaCl buffer (0.01% SDS, 10 mM PBS) was added every 4 h with brief sonication for a total of 24 h. Excess AS1411 and anti221 was removed by centrifugation at 4500 rpm for 20 min to obtain the NPsFA-AS1411/a221.

For DOX loading, 1 mg/mL of free DOX was added to the NPsFA-AS1411/a221 solution, and the mixture was stirred for 12 h. The precipitate was separated by centrifugation and washed with deionized water several times until the supernatant became colorless. NPsFA-AS1411/DOX/a221 was collected and suspended in 10 mM PBS buffer. NPsFA-AS1411/DOX/a221 was characterized by UV-vis spectroscopy (Shimadzu UV-1800) and DLS.

The standard curve method was used to determine the AS1411, anti221 and DOX loading capacities per NPs^23^. The fluorescence intensity at different concentrations of FITC- and Cy3- labeled AS1411 or anti-221 and DOX was measured using a Shimadzu RF-5301PC fluorescence spectrophotometer.

### Cell culture and transfection

K562 (parental cells), drug-resistant K562 (K562DR) and LO2 cells were maintained in RPMI1640 cell medium containing 10% fetal bovine serum (FBS) (Life Technology) at 37 °C under a 5% CO_2_ atmosphere. Cells (1 × 10^6^) were seeded onto 6-well plates overnight before transfection. 100 nM of siRNA oligos and scrambled controls were introduced into cells using Lipofectamine™ RNAiMAX (Life Technologies).

### Specific targeting of K562DR cells

Specific targeting of K562DR cells was determined by fluorescence and dark-field imaging. NPsFA-AS1411/DOX/a221 was added to 562DR cells followed by incubation for 6 h at 37 °C. The cells were collected by centrifugation at 1000 rpm for 10 min, washed with PBS and placed on a slide. The cells were fixed with 4% formaldehyde for 10 min and stained with Hoechst 33342 for another 10 min. The cells were washed three times before images were taken. The control groups were subjected to the same process.

### Drug released from NPsFA-AS1411/DOX/a221 in cancer cells

NPsFA-AS1411/DOX/a221 and free DOX (the DOX concentration being equal) were added to K562DR cells followed by incubation for 4 h, 8 h and 12 h. Cells were then washed with PBS and stained with Hoechst 33342 to highlight the cell nuclei. Fluorescence imaging was performed using an Olympus confocal microscope.

### Colony-forming, cell proliferation and apoptosis assays

Colony-forming assays were performed in MethoCult® mixture (Stem Cell Technologies). Apoptosis was measured using the Annexin V-FITC/PI method. Cell proliferation was measured using the Cell Counting Kit-8 (CCK8, Dojindo Molecular Technologies), following the manufacturer’s instructions. Microplates were incubated at 37 °C for 24 hours. Absorbance was measured at 450 nm using a microplate reader. Reported IC50 values represent the mean of three independent experiments performed in quadruplicate.

### Western blotting

Western blotting was performed as previously described^41^. Briefly, whole cellular lysates were prepared by harvesting the cells in cell lysis buffer. The antibodies used were purchased from Santa Cruz Biotechnology (anti-DNMT1, anti-P-gp), Cell Signaling Technology (anti-p27kip1), Abcam (anti-nucleolin) and Sigma Aldrich (anti-β-actin).

### Quantitative PCR (qPCR)

RNA preparation and qPCR were performed as previously described^42^. Briefly, total RNA was isolated using the miRNAeasy kit (Qiagen) and cDNA synthesis was performed using High Capacity cDNA Reverse Transcription Kits (Applied Biosystems). qPCR was performed using the SYBR-Green master mix (Applied Biosystems). The levels of 18S were used for normalization and the results were analyzed using the ΔCT approach. The primer sequences are listed in Table 2.

**Table 2:** Sequences of primers used in the experiments.

Name		Primer sequence (5′ to 3′)
AS1411	SH-	HS-TTTTTTTTTTCGTCGTCGTCGTCGTCGTCGTCGT
Anti-221	transfection	GAAACCCAGCAGACAATGTAGCT
	SH-	SH-TTTTTTTTTTTGAAACCCAGCAGACAATGTAGCT
CRO26	transfection	CCTCCTCCTCCTTCTCCTCCTCCTCC
	SH-	SH-TTTTTTTTTTTCCTCCTCCTCCTTCTCCTCCTCCTCC
*p15* ^*INK4B*^ *(human)*	forward	CCAGATGAGGACAATGAG
	reverse	AGCAAGACAACCATAATCA
*P27kip1 (human)*	forward	GAAGCAAGGAAGATATACAT
	reverse	CACAGGTAGTACAATGAAG
*NCL*	forward	AAGGCACAGAACCGACTA
	reverse	GACATCCACAACAGCAAGA
*DNMT1(human)*	forward	AGATGACGATGAGGAAGT
	reverse	ATGCGATTCTTGTTCTGT
*18S (human)*	forward	ACAGGATTGACAGATTGA
	reverse	TATCGGAATTAACCAGACA

### Leukemia patient samples

Gene Expression Omnibus (GEO) datasets (GSE68466 and GSE68467^43^) were analyzed for the mRNA expression of miR-221 and P-gp, which was assessed by gene expression arrays (109 leukemia patients). The detailed clinical characteristics of the patients can be consulted in the original reports. Data were normalized, managed and analyzed by means of Spearman correlation coefficients using GraphPad Prism 6 software.

Bone marrow evaluation for the diagnosis of AML was performed according to the criteria of the World Health Organization. The peripheral blood from AML patients (n = 3) was obtained from the Tumor Tissue/Biospecimen Bank of the First Hospital of Jilin University and mononuclear cells were purified using Ficoll-PaqueTM PLUS (GE Healthcare). The primary cells were handled and cultured as previously described^44^. The human study was approved by the Institutional Review Board of the First Hospital of Jilin University. All patients signed an informed consent document approved by the Institutional Review Board before entering the study. The patients’ information is presented in Table 1.

### Statistical analysis

Quantified target changes were analyzed using the Student’s t-test. Correlations were determined by means of Pearson correlation coefficients. Differences were considered statistically significant at *P* < 0.05. All p-values were calculated by using the unpaired, two-tailed Student’s t-test.

## Supplementary information

SUPPLEMENTARY INFO

## References

[1] Cersosimo RJ, Hong WK (1986). Epirubicin: a review of the pharmacology, clinical activity, and adverse effects of an adriamycin analogue. J Clin Oncol.

[2] Gottesman MM, Fojo T, Bates SE (2002). Multidrug resistance in cancer: role of ATP-dependent transporters. Nat Rev Cancer.

[3] O’Hare T (2009). AP24534, a pan-BCR-ABL inhibitor for chronic myeloid leukemia, potently inhibits the T315I mutant and overcomes mutation-based resistance. Cancer Cell.

[4] Lee HJ (2014). Drug Resistance via Feedback Activation of Stat3 in Oncogene-Addicted Cancer Cells. Cancer Cell.

[5] Brown R, Curry E, Magnani L, Wilhelm-Benartzi CS, Borley J (2014). Poised epigenetic states and acquired drug resistance in cancer. Nat Rev Cancer.

[6] Yan F (2015). The DNA Methyltransferase DNMT1 and Tyrosine-Protein Kinase KIT Cooperatively Promote Resistance to 5-Aza-2′-deoxycytidine (Decitabine) and Midostaurin (PKC412) in Lung Cancer Cells. J Biol Chem.

[7] Karp JE (2001). MDR modulation in acute myelogenous leukemia: is it dead?. Leukemia.

[8] Robey, R. W. *et al*. Revisiting the role of ABC transporters in multidrug-resistant cancer. *Nat Rev Cancer*, 10.1038/s41568-018-0005-8 (2018). 10.1038/s41568-018-0005-8 PMC6622180 29643473

[9] Eadie LN (2017). The clinical significance of ABCB1 overexpression in predicting outcome of CML patients undergoing first-line imatinib treatment. Leukemia.

[10] Maeda H, Nakamura H, Fang J (2013). The EPR effect for macromolecular drug delivery to solid tumors: Improvement of tumor uptake, lowering of systemic toxicity, and distinct tumor imaging *in vivo*. Adv Drug Deliv Rev.

[11] Ye, M. Z. *et al*. A Tumor-Specific Cascade Amplification Drug Release Nanoparticle for Overcoming Multidrug Resistance in Cancers. *Adv Mater* **29**, 10.1002/adma.201702342 (2017). 10.1002/adma.201702342 28833669

[12] Shen, W. *et al*. A glutathione-responsive sulfur dioxide polymer prodrug as a nanocarrier for combating drug-resistance in cancer chemotherapy. *Biomaterials*, 10.1016/j.biomaterials.2018.02.011 (2018). 10.1016/j.biomaterials.2018.02.011 29433753

[13] Zhang Y (2017). Overcoming Tamoxifen Resistance of Human Breast Cancer by Targeted Gene Silencing Using Multifunctional pRNA Nanoparticles. Acs Nano.

[14] Dawson KA, Yan Y (2016). Drug delivery: Leukocyte-like carriers. Nat Mater.

[15] Parodi A (2013). Synthetic nanoparticles functionalized with biomimetic leukocyte membranes possess cell-like functions. Nat Nanotechnol.

[16] Huang X (2013). Targeted delivery of microRNA-29b by transferrin-conjugated anionic lipopolyplex nanoparticles: a novel therapeutic strategy in acute myeloid leukemia. Clinical cancer research: an official journal of the American Association for Cancer Research.

[17] Yan F (2018). A vicious loop of fatty acid-binding protein 4 and DNA methyltransferase 1 promotes acute myeloid leukemia and acts as a therapeutic target. Leukemia.

[18] Shen N (2018). HDL-AuNPs-BMS Nanoparticle Conjugates as Molecularly Targeted Therapy for Leukemia. ACS applied materials & interfaces.

[19] Ganju A (2017). miRNA nanotherapeutics for cancer. Drug Discov Today.

[20] Adams BD, Parsons C, Walker L, Zhang WC, Slack FJ (2017). Targeting noncoding RNAs in disease. The Journal of clinical investigation.

[21] Gulla A (2016). A 13 mer LNA-i-miR-221 Inhibitor Restores Drug Sensitivity in Melphalan-Refractory Multiple Myeloma Cells. Clinical cancer research: an official journal of the American Association for Cancer Research.

[22] Fornari, F. *et al*. In Hepatocellular Carcinoma miR-221 Modulates Sorafenib Resistance through Inhibition of Caspase-3-Mediated Apoptosis. *Clinical cancer research: an official journal of the American Association for Cancer Research*, 10.1158/1078-0432.CCR-16-1464 (2017). 10.1158/1078-0432.CCR-16-1464 28096271

[23] Deng R (2018). Targeting epigenetic pathway with gold nanoparticles for acute myeloid leukemia therapy. Biomaterials.

[24] Lynn RC (2015). Targeting of folate receptor beta on acute myeloid leukemia blasts with chimeric antigen receptor-expressing T cells. Blood.

[25] Peng Y (2017). Smart Human-Serum-Albumin-As2 O3 Nanodrug with Self-Amplified Folate Receptor-Targeting Ability for Chronic Myeloid Leukemia Treatment. Angewandte Chemie.

[26] Zhou W, Gao X, Liu DB, Chen XY (2015). Gold Nanoparticles for *In Vitro* Diagnostics. Chemical Reviews.

[27] Li LY (2014). Nucleolin-targeting liposomes guided by aptamer AS1411 for the delivery of siRNA for the treatment of malignant melanomas. Biomaterials.

[28] Liao ZX (2015). An AS1411 aptamer-conjugated liposomal system containing a bubble-generating agent for tumor-specific chemotherapy that overcomes multidrug resistance. J Control Release.

[29] Han G (2005). Controlled recovery of the transcription of nanoparticle-bound DNA by intracellular concentrations of glutathione. Bioconjug Chem.

[30] Su S (2013). Design and applications of gold nanoparticle conjugates by exploiting biomolecule-gold nanoparticle interactions. Nanoscale.

[31] Kedde M (2010). A Pumilio-induced RNA structure switch in p27-3′ UTR controls miR-221 and miR-222 accessibility. Nat Cell Biol.

[32] Garofalo M (2009). miR-221&222 regulate TRAIL resistance and enhance tumorigenicity through PTEN and TIMP3 downregulation. Cancer Cell.

[33] Fletcher JI, Haber M, Henderson MJ, Norris MD (2010). ABC transporters in cancer: more than just drug efflux pumps. Nature Reviews Cancer.

[34] Shang Y (2014). miR-508-5p regulates multidrug resistance of gastric cancer by targeting ABCB1 and ZNRD1. Oncogene.

[35] Zhao XH, Yang L, Hu JG, Ruan JG (2010). miR-138 might reverse multidrug resistance of leukemia cells. Leukemia research.

[36] Lee J, Choi KJ, Moon SU, Kim S (2016). Theragnosis-based combined cancer therapy using doxorubicin-conjugated microRNA-221 molecular beacon. Biomaterials.

[37] Shiao YS, Chiu HH, Wu PH, Huang YF (2014). Aptamer-Functionalized Gold Nanoparticles As Photoresponsive Nanoplatform for Co-Drug Delivery. ACS applied materials & interfaces.

[38] Xiang DX (2015). Nucleic Acid Aptamer-Guided Cancer Therapeutics and Diagnostics: the Next Generation of Cancer Medicine. Theranostics.

[39] Rosenberg JE (2014). A phase II trial of AS1411 (a novel nucleolin-targeted DNA aptamer) in metastatic renal cell carcinoma. Investigational new drugs.

[40] Ji X (2007). Size control of gold nanocrystals in citrate reduction: the third role of citrate. J Am Chem Soc.

[41] Yan F (2017). A regulatory circuit composed of DNA methyltransferases and receptor tyrosine kinases controls lung cancer cell aggressiveness. Oncogene.

[42] Yan F (2017). Fatty acid-binding protein FABP4 mechanistically links obesity with aggressive AML by enhancing aberrant DNA methylation in AML cells. Leukemia.

[43] Chiu YC (2016). Prognostic significance of NPM1 mutation-modulated microRNA - mRNA regulation in acute myeloid leukemia. Leukemia.

[44] Gao XN (2015). AML1/ETO cooperates with HIF1 alpha to promote leukemogenesis through DNMT3 alpha transactivation. Leukemia.

