# Supplementary material for: Multifunctional Gold Nanoparticles Overcome MicroRNA Regulatory Network Mediated-Multidrug Resistant Leukemia
Source: Sci Rep. 2019 Mar 29;9:5348. doi: 10.1038/s41598-019-41866-y (PMC6440980; doi:10.1038/s41598-019-41866-y)
Supplement: Supplementary file 1 — SUPPLEMENTARY INFO [file 41598_2019_41866_MOESM1_ESM.pdf]

Supporting Information

Multifunctional Gold Nanoparticles Overcome MicroRNA Regulatory Network Mediated-Multidrug Resistant Leukemia

Rong Deng[a,b], *Bai Ji*[c], *Hongliang Yu*[b], *Wei Bao*[a], *Zhuoqi Yang*[b], *Ying Yu*[a], *Yahan Cui*[b], *Yangyang Du*[a], *Meiyu Song*[a], *Shujun Liu*[d], *Kamel Meguellati*[b,*], *Fei Yan*[a,b,*]

[a]State Key Laboratory of Inorganic Synthesis and Preparative Chemistry, International Research Center for Chemistry-Medicine Joint Innovation, College of Chemistry, Jilin University, 2699 Qianjin Street, Changchun 130012, China

[b]International Joint Research Laboratory of Nano-Micro Architecture Chemistry (NMAC), College of Chemistry, Jilin University, 2699 Qianjin Street, Changchun 130012, China

[c]Department of Hepatobiliary and Pancreatic Surgery, the First Hospital of Jilin University, Changchun 130021, China.

[d]The Hormel Institute, University of Minnesota, 801 16th Avenue NE, Austin, MN 55912, USA;

Correspondence and requests for materials should be addressed to F. Y. (email: feiyan@jlu.edu.cn) or to K. M. (email: kameljilin@yahoo.fr).

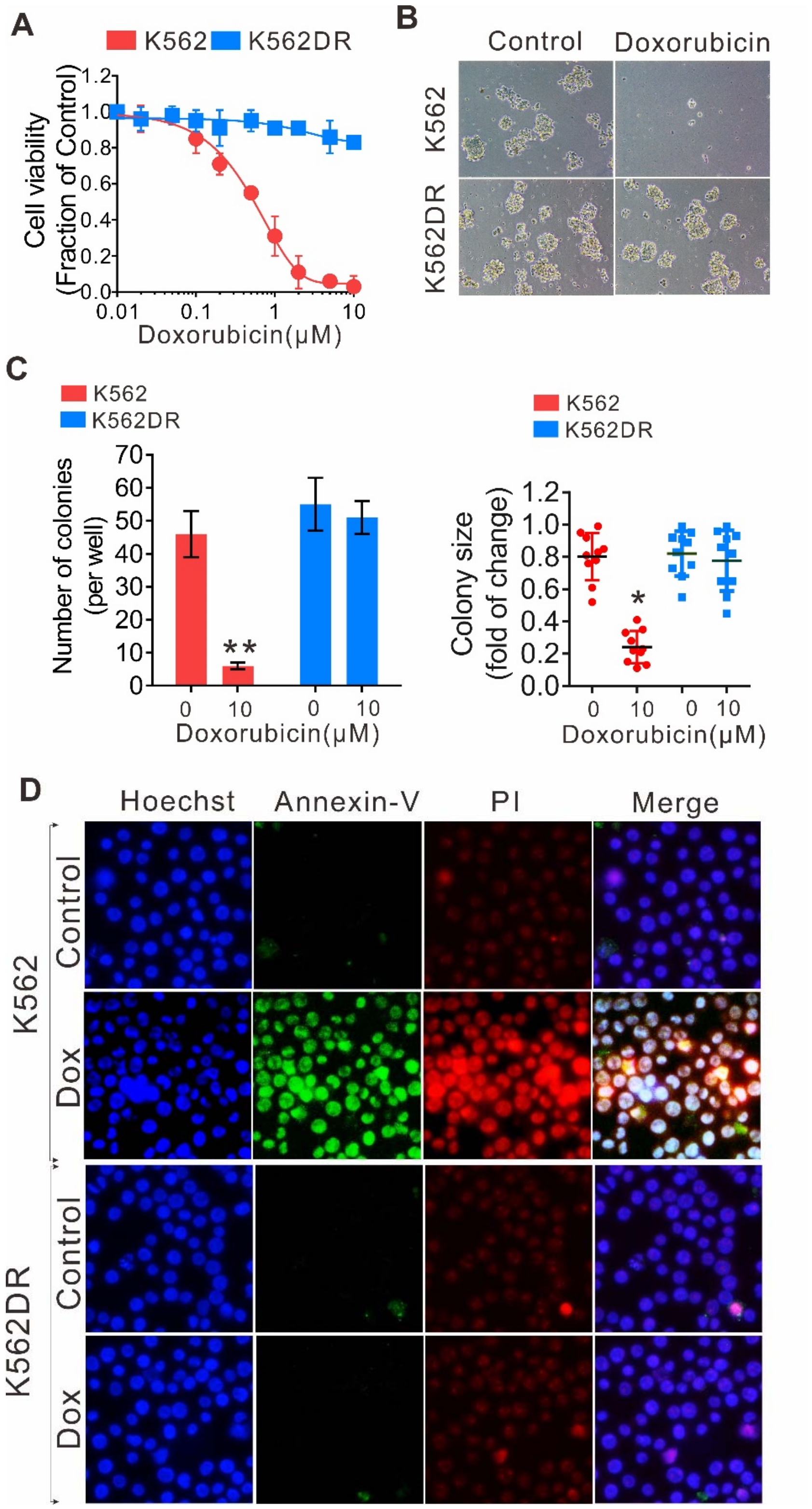

Figure S1. Characterization of K562 drug-resistant cells. (A) CCK-8 assays for cell proliferation in parental K562 and resistant cells treated with doxorubicin for 72 hours. The data represent three independent experiments with three repeats in total. (B) Colony-forming assays in parental cells treated with 10 μM doxorubicin or PBS control. Photographs show representative clones. (C)

Graphs indicate the colony number from 3 independent experiments (left) and quantification of clone sizes from ten clones (right). Data are mean ±SD; *$P<0.05$, **$P<0.01$. (D) Fluorescence assay for apoptosis in K562 or K562DR cells treated with 10 μM doxorubicin or PBS control for 24 hours (blue, hoechst; green, Annexin-V; red, PI).

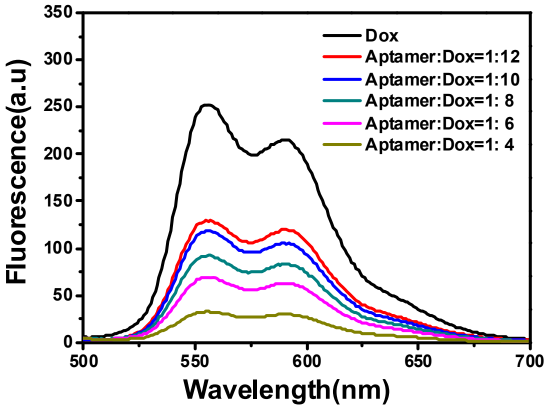

Figure S2. Flourescence for the interaction between DOX and AS1411. Fluorescence spectra of Dox when they were conjugated with AS1411 under different molar ratios.

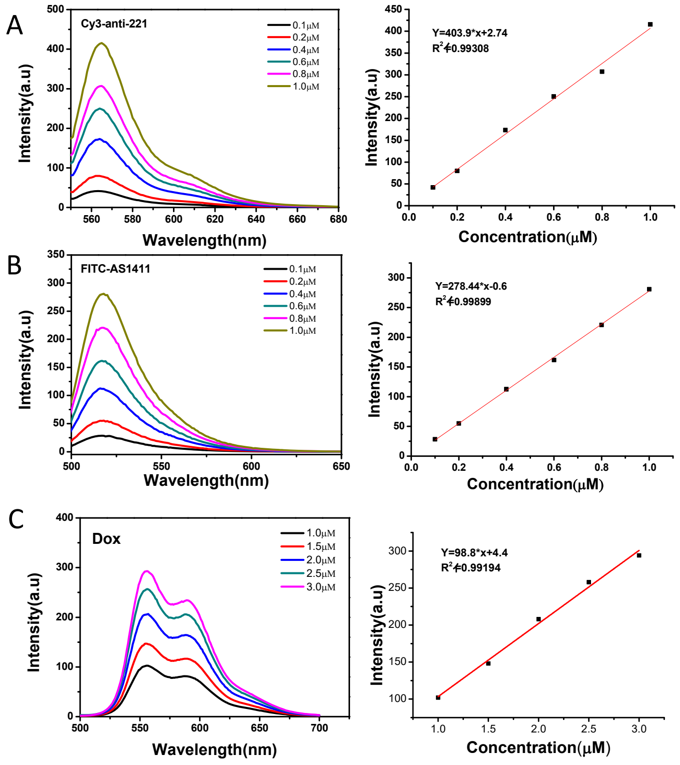

D

Type / Measurement	AS1411	anti221	Dox
1	103/200	101/200	284/500
2	106/200	95/200	292/500
3	112/200	106/200	281/500
SEM	4.6	5.5	5.7
Average	107	101	286

Figure S3. Estimation of the loading capacity of AS1411, anti-221 and DOX on the surface of gold nanoparticles. (A-C) Fluorescence spectra and standard curves of AS1411, anti-221 and DOX in the PBS buffer. (D) Summary of the loading number of AS1411, anti-221 and DOX on the surface of gold nanoparticles (mol per particle).

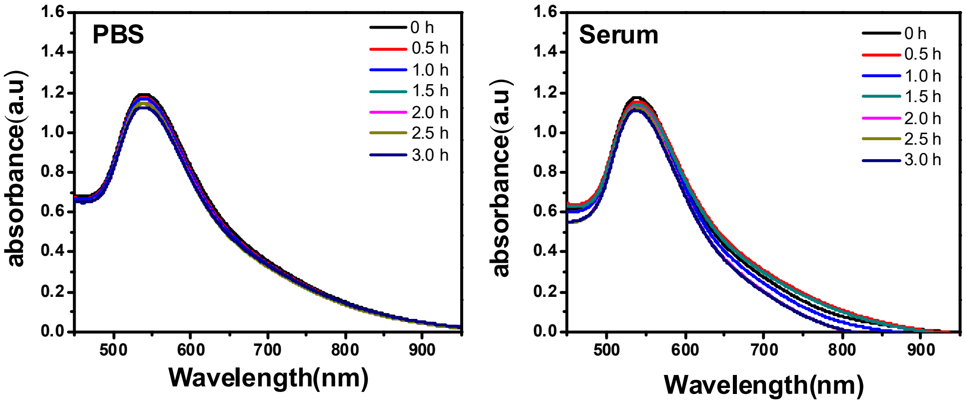

Figure S4. The stability of the NPsFA- AS1411/DOX/a221 in PBS and serum for 3 h.

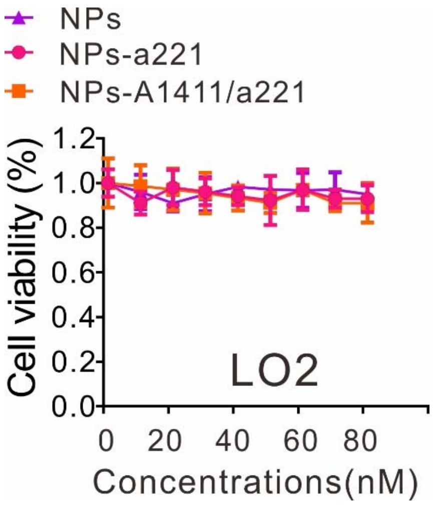

Figure S5. Nanoparticles have no effect on LO2 cells. CCK-8 assays for cell proliferation in LO2 cells treated with different doses of indicated nanoparticles for 72 hours. The data represent 3 independent experiments with 3 repeats in total.

Full-length gels

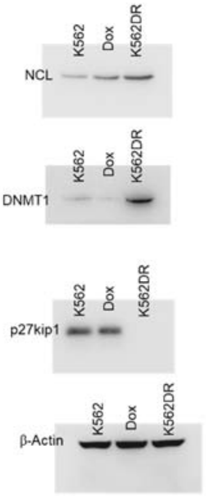

Fig 2D

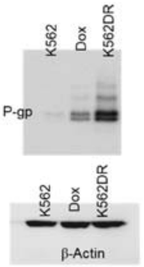

Fig 2F

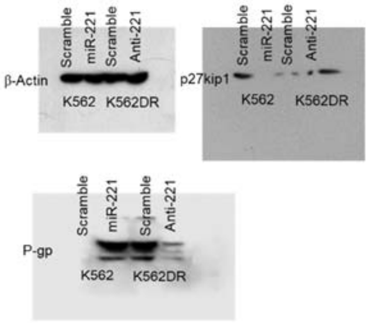

Fig 2H

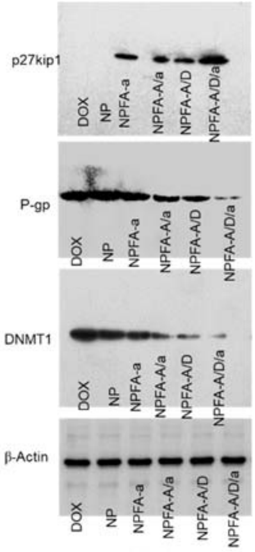

Fig 5C
